# ACL Repair: A Game Changer or Will History Repeat Itself? A Critical Appraisal

**DOI:** 10.3390/jcm10050912

**Published:** 2021-02-26

**Authors:** Christiaan H. W. Heusdens

**Affiliations:** Department of Orthopedics, Antwerp University Hospital, 2650 Edegem, Belgium; Krik.Heusdens@uza.be; Tel.: +32-3-821-5483

**Keywords:** anterior cruciate ligament, ACL repair, dynamic intraligamentary stabilization, suture tape augmentation, suture tape reinforcement, suture anchor primary repair, bridge-enhanced ACL repair

## Abstract

Until the past decade the common thought was that the anterior cruciate ligament (ACL) was not able to heal and restore knee stability. In this manuscript a brief review of studies of the developers and the early adaptors of four different modern ACL repair techniques are presented. The present status and considerations for the future of ACL repair and its research are shared. After promising short- to midterm ACL healing results by the developers, the results of the early adaptors show more variety in terms of rerupture and reintervention for other reasons. Risk factors for failure are a young age, high preinjury sports activity level, midsubstance ruptures and impaired integrity of the ACL bundles and the synovial sheath. There is a call for more clinical data and randomized clinical trials. Conclusion: an important finding of the past decade is that the ACL is able to heal and subsequently restabilize the knee. Patient selection is emphasized: the ideal patient is a non-high athlete older than 25 and has an acute proximal one bundle ACL rupture. Further research will have to show if ACL repair could be a game changer or if history will repeat itself.

## 1. Introduction

Anterior cruciate ligament (ACL) repair returned to the spotlight with the introduction of modern ACL repair techniques. Although initially good short-term results after open ACL repair were presented in the 1970s, midterm results deteriorated. Feagin et al. reported a significant reinjury rate after repair of the ACL in 17 out of 32 patients treated with an open repair and five-year follow-up [1]. The technique used for open ACL repair consisted of an arthrotomy, suturing of the ACL with drill holes in the femur and cast immobilization for 4–6 weeks [2]. This open repair technique was replaced by arthroscopic ACL reconstruction (ACL recon) in the 1980s.

ACL recon is the gold standard for surgical treatment of the ruptured ACL despite a number of problems related to this surgery: anterior knee pain (20%), kneeling pain (15%), hamstring muscle weakness following harvesting (10%), rotatory instability with a positive pivot shift (24%), rerupture (6%, up to 28% in high-risk populations), and clinical failure (10%), and only 50% to 65% of recreational athletes return to their preinjury level of sports [3,4,5]. Another disadvantage of conventional ACL recon is the extensive rehabilitation period. On average, patients return to their work after 11 weeks and are allowed to return to sports after 9–12 months [6]. ACL recon has a huge socio-economic impact, as the majority of ACL injuries occur in people of working age [6,7]. ACL reconstructed knees and nonoperatively treated knees demonstrated a risk of 4.71 times and 2.41 times, respectively, for development of moderate to severe arthritis compared with controls [8]. In a prospective study of 958 patients treated with bone–patellar tendon–bone or hamstring–tendon graft ACL recon with two years of follow-up, the total rate of complications was 39% and the surgical revision rate for any reason was 28% [3]. Given the limitations and risks associated with the current gold standard treatment of an ACL rupture, there is room for improvement.

It was common thought that the ACL was not able to heal and restore knee stability, until Costa-Paz et al. and Steadman et al. documented the healing of the ACL in 2012 [9,10]. In the past decade, four different modern ACL repair techniques have been introduced. ACL repair could be a promising surgical technique with theoretical advantages over ACL recon. Modern ACL repair techniques are less invasive compared to ACL recon. If bone tunnels are drilled for the repair techniques, they are less than half the size of the bone tunnels needed for ACL recon. There is no graft harvesting morbidity as no graft is needed. Preservation of the native ACL ligament and its proprioceptors contributes in the feedback on position and dynamic stability of the knee, which could reduce the rehabilitation period and therefore the economic burden [11]. ACL repair has the potential to preserve the native insertion site as well, which may in turn lead to more normal joint mechanics and decreased risk of post-traumatic osteoarthritis [12]. Another advantage is that in the event of a rerupture, a standard ACL recon can be performed. “No bridges are burned.” The author started with ACL repair in 2014 as an early adaptor and has performed more than 130 ACL repairs with three of the four ACL repair techniques. In this manuscript, a brief review of studies of the developers and the early adaptors of four different modern ACL repair techniques are presented. The present status and considerations for the future of ACL repair and its research are shared.

## 2. Literature by the Developers

### 2.1. Dynamic Intraligamentary Stabilization

**Figure 1:**
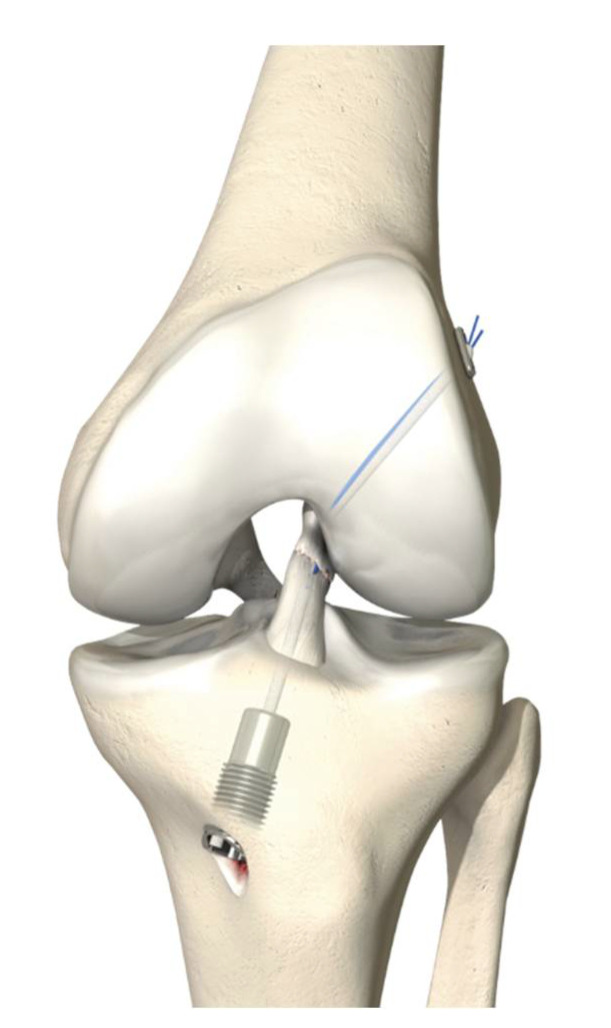
Dynamic intraligamentary stabilization of the left knee, frontal view. This image can be found in the “Surgical technique Ligamys” brochure, Mathys Ltd. Bettlach [3]. Permission was granted by the company, Mathys Ltd. Bettlach, to use this picture in a journal article.

In 2012, Sandro Kohl et al. published an animal study of a new ACL repair technique, dynamic intraligamentary stabilization (DIS, Ligamys, Mathys Ltd., Bettlach, Switzerland) [13]. The ruptured ACL is brought back to its origin with polydioxanone sutures (PDS) and the knee is stabilized with a strong suture alongside the ACL, which is fixed in the tibia with a spring–screw system (Figure 1). In 2014, Sandro Kohl et al. describe a potential biomechanical solution for the ACL repair failures in the past [14]. A rigid fixation was used to repair the ACL, which failed upon cyclic loading. By creating a dynamic fixation that restored anteroposterior (AP) stability and could withstand the repetitive cyclic forces, a biomechanically stable environment was created in which the ACL could heal [14]. The next year, the results of the first 10 patients treated with DIS with a two-year follow-up were reported [15]. This treatment resulted in stable clinical and radiological healing of the torn ACL in all but one patient of this first series. They attained normal knee scores, reported excellent satisfaction and could return to their previous level of sporting activity. A case series of 278 patients treated with DIS for an acute ACL rupture with a mean follow-up of 14 months showed noninferior patient-reported outcome measures (PROMs) compared to preoperatively, stable AP knees and a rerupture rate of 2.9% [16]. In summary, promising results of a novel treatment for acute ACL repair were presented by the developers of the DIS technique at the end of 2014. In 2016, Kohl et al. reported a high rate of secondary interventions in a group of 50 patients with a two-year follow-up. In that case, 10% developed instability, 10% needed an arthrofibrolysis, and 60% required removal of the tibial screw [17].

### 2.2. Suture Tape Augmentation/Internal Brace Ligament Augmentation

**Figure 2:**
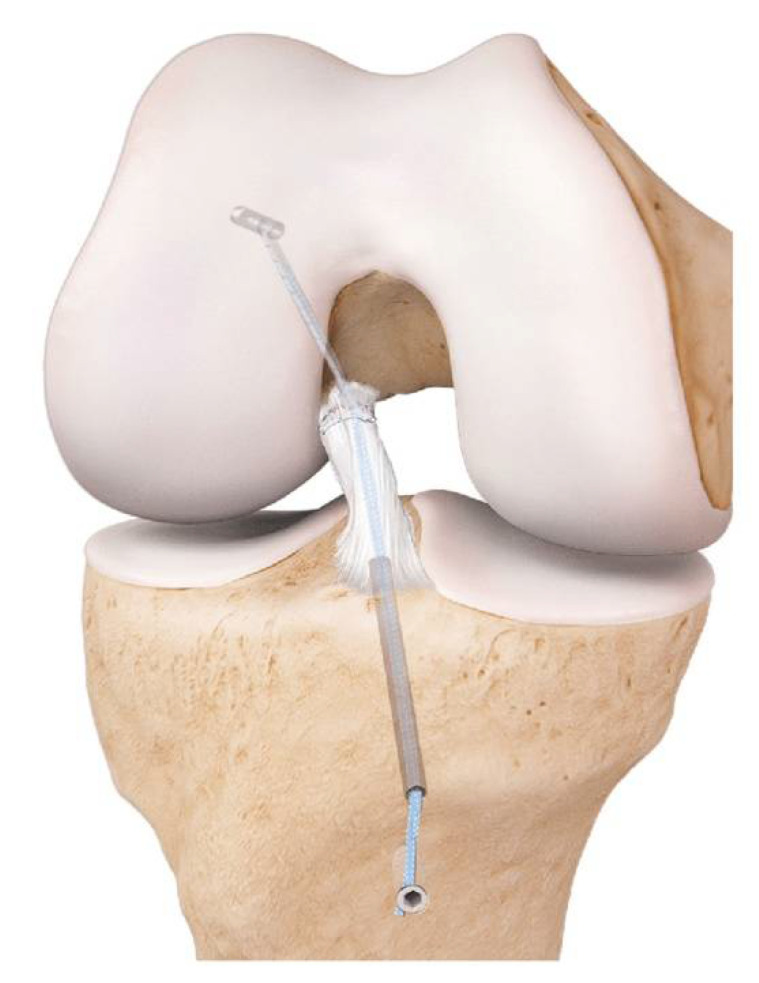
Internal brace ligament augmentation of the right knee, frontal view. This image can be found in the “ACL Primary Repair with *Internal*Brace Surgical technique” brochure, Arthrex GmbH [22]. Permission was granted by the company, Arthrex GmbH, to use this picture in a journal article.

The suture tape augmentation (STA) technique, also called suture tape reinforcement or internal bracing ligament augmentation technique (*Internal*Brace, Arthrex GmbH, Naples, FL, USA) is a repair technique that can be used for all knee ligaments, including the ACL, and for ankle, elbow and shoulder ligaments as well. The ruptured parts of the ACL are brought together with a lasso suture and protected with a 2 mm wide high-strength tape that acts as an internal brace to provide an environment in which the ACL can heal (Figure 2) [18]. This internal brace reinforces the ligament as a secondary stabilizer, encouraging natural healing of the ligament by protecting it during the healing phase and supporting early mobilization. Heitmann et al. published in 2014 a biomechanical study on porcines. In this study, the augmented suture repair of the ACL provides significantly higher stability compared with isolated suture repair or reconstruction with hamstring tendons [19]. MacKay et al. published in 2015 a review on ligament augmentation with the internal bracing technique containing the one-year follow-up results of 68 patients [20]. The results of this study suggest that at short-term follow-up, repair with the STA technique is at least as effective in restoring stability and function to the knee as traditional ACL recon surgery. Two-year follow-up results of 42 patients treated with the STA technique by the developer showed that a meaningful Knee Injury and Osteoarthritis Outcome Score (KOOS) sport and recreation change and significant improvements in the KOOS Visual Analogue Pain Scale (VAS pain), Veterans RAND 12-item health survey (VR-12) physical scores as well as a significant decrease of the Marx activity scale in comparison to preoperative scores are demonstrated [21]. Two of the 42 patients (4.8%) reported an ACL rerupture. They conclude that repair with this technique could be clinically relevant as a treatment option for patients with an acute, proximal ACL rupture that is not retracted and is of good tissue quality.

### 2.3. Suture Anchor Primary ACL Repair

**Figure 3:**
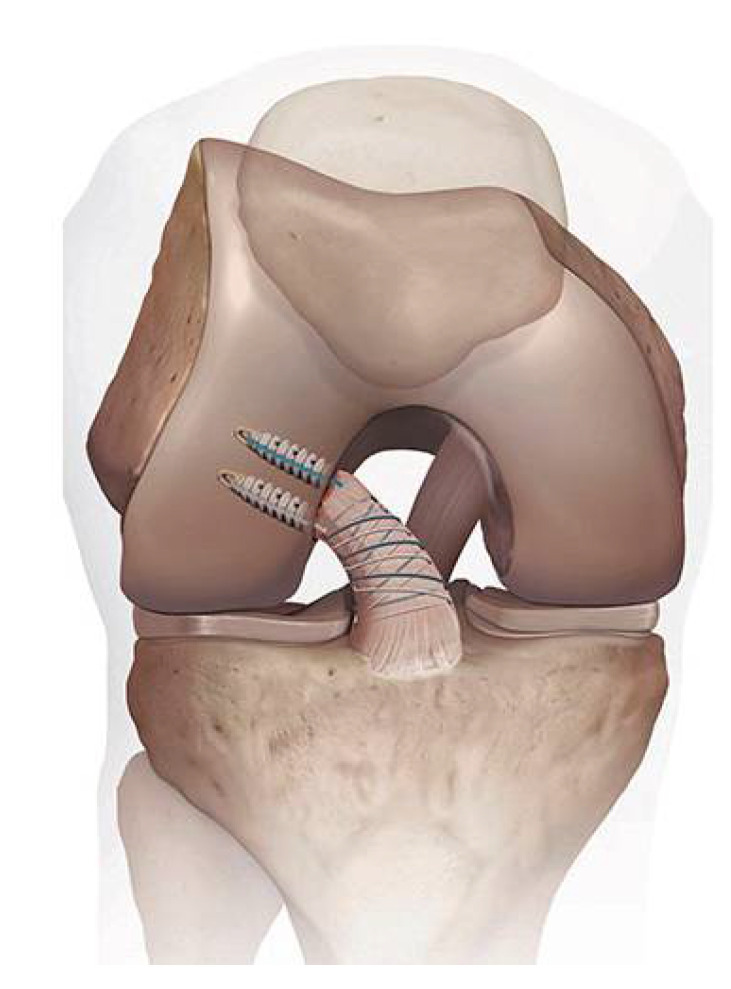
Suture anchor primary anterior cruciate ligament (ACL) repair of the right knee, frontal view. This image can be found in the “ACL Primary Repair Surgical Technique” brochure, Arthrex GmbH [33]. Permission was granted by the company, Arthrex GmbH, to use this picture in a journal article.

Difelice et al. published in 2015 the results of an early follow-up of 11 consecutive cases treated with suture anchor primary repair (SAPR) of the ACL with a mean follow-up of 3.5 years [23]. For the SAPR technique, the ruptured ACL was sutured starting at the intact distal end of the ligament and advanced in an alternating, locking Bunnell-type pattern up to the avulsed end for both the anteromedial and posterolateral bundle. Sutures were fixed with a suture anchor at the anteromedial and posterolateral femoral origin site of the ACL (Figure 3). In their study, one patient had a rerupture and one patient had a KT-1000 AP laxity side-to-side difference of 6 mm. They concluded that this technique can achieve short-term clinical success in a carefully selected subset of patients with proximal avulsion tears and excellent tissue quality [23]. These clinical outcomes were maintained at a mean follow-up of 6.0 ± 1.5 years [24]. In the following years, Difelice and van der List have performed extensive work on modern ACL repair. They proposed a treatment algorithm for ACL injuries that is based on tear location and tissue quality [25,26]. A retrospective study on 52 repairs and 90 reconstructions showed that following primary repair, patients had better range of motion and trends towards fewer complications than with reconstruction [27]. In a cohort study, 56 consecutive patients underwent arthroscopic ACL SAPR, of which the latter 27 patients (48.2%) received internal bracing in addition to ACL SAPR. They reported good objective and subjective outcomes at a 3.2-year follow-up in a carefully selected population, with a failure rate of 7.4% for patients treated with ACL SAPR with internal bracing and 13.8% for patients without internal bracing. There were no statistically significant or clinically relevant differences in subjective outcomes [28]. In a large cohort study, it was noted that 44% of patients with an ACL rupture had repairable ACL tears. Primary repair was more likely to be possible in older patients, patients with lower BMI and when surgery was performed within four weeks of injury [29]. Treatment failure was found to be significantly higher in the age group <22 years (37.0%) as compared to the 22–35 years (4.2%) and the >35 years (3.2%) groups [30]. Different studies showed that tear location and tissue quality on preoperative MRI can predict eligibility for arthroscopic primary ACL repair, and postoperative MRI was found to accurately predict the chance of rerupture of the primarily repaired ACL [30,31,32].

### 2.4. Bridge-Enhanced ACL Repair

**Figure 4:**
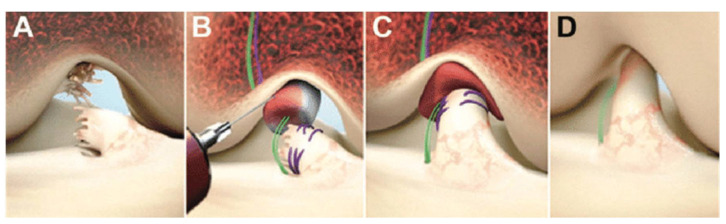
Bridge-enhanced anterior cruciate ligament (ACL) repair, frontal view right knee: (**A**) Ruptured ACL; (**B**) The scaffold is saturated with the patient’s blood; (**C**) The tibial stump is pulled up into the saturated scaffold; (**D**) Healing of the ACL [40].

Compared to the previously mentioned repair techniques, extensive fundamental research and animal studies have been published on the bridge-enhanced ACL repair (BEAR) technique [22,33,34,35,36,37,38]. The BEAR technique involves suture repair of the ligament combined with a bioactive scaffold to bridge the gap between the torn ligament ends (Figure 4). In 2016, the first-in-human cohort study compared BEAR with ACL recon, and outcomes were assessed three months postoperatively. The results of this study suggested that the BEAR procedure had a rate of adverse reactions low enough to warrant a study of efficacy in a larger group of patients [39]. At two years’ follow-up, there were no graft or repair failures, and BEAR produced similar outcomes to ACL recon with a hamstring autograft [40]. A randomized controlled trial (RCT) of 65 BEAR versus 35 ACL recon patients showed similar outcomes in both treatment groups for PROMs and AP knee laxity two years postoperatively in a young and active cohort [34]. Reinjuries that required a second ipsilateral ACL surgical procedure occurred in 14% of the BEAR group and 6% of the ACL recon group. Eight of the patients that converted from BEAR to ACL recon in the study period had similar primary outcomes to patients who had a single ipsilateral ACL procedure [34].

## 3. Early Adaptor Phase

### 3.1. Dynamic Intraligamentary Stabilization

Most ACL repair studies by early adaptors have been published on DIS. After promising short- to midterm results by the developers, the DIS results by the early adaptors show more variety. Five years’ follow-up has been reported by Kösters et al. on 65 patients treated with DIS [41]. Eight patients (12.3%) had a rerupture, and four (6.2%) patients had to be revised performing an arthrolysis due to extension deficit. Ahmad et al. report a minimum five years’ survival rate after primary ACL DIS repair of 70% [42]. This value dropped to 56% in highly active patients performing competitive sports. Patients not suffering failure of repair demonstrated adequate restoration of knee laxity and high satisfaction. Several short-term case reports showed a failure rate of 15% or more and a high resurgery rate for other reasons than revision [43,44,45,46,47]. Other short-term case reports confirm the positive results of the developers group with an ACL failure of less than 10% and a low resurgery rate for other reasons than revision [46,48,49]. Ateschrang et al. performed an arthroscopy on 47 patients treated with DIS after a minimum postoperative interval of six months for semiquantitative evaluation of ACL integrity, function and scar-tissue formation [50]. Full restoration of the ACL volume was affirmed in 30 (63.8%) patients and two-thirds restoration in 13 (27.7%). Hypertrophic scar formation was observed in 23 (48.9%) patients. Deficient ACL recovery was noted in four patients (8.5%), of which no one required secondary reconstructive surgery. Two RCTs have been published. Hoogeslag et al. randomized 48 patients who underwent DIS (24 patients) or ACL recon (24 patients) [51]. In the DIS group, 8.7% experienced a rerupture and 20.8% were treated with repeat surgeries versus 19% reruptures and 12.5% repeat surgeries in the ACL recon group. DIS was not inferior in terms of an International Knee Documentation Committee (IKDC) subjective score two years postoperatively. Kösters et al. randomized 85 patients between DIS (43 patients) and ACL recon (42 patients) [52]. A total of seven patients (16.3%) in the DIS group experienced clinical failure and underwent single-stage revision. In the ACL recon group, five patients (12.5%) experienced failure of the reconstruction procedure; four (10%) of these patients required 2-stage revision. Anterior tibial translation measured by Rolimeter testing was significantly increased after ACL repair with DIS, whereas clinical failure was similar to that after ACL recon. In addition, functional results after ACL repair with DIS for acute tears were comparable with those after ACL recon. Risk factors described for failure after DIS repair are: young age, high preinjury sports activity level, high knee laxity, midsubstance ruptures, and impaired integrity of the ACL bundles and the synovial sheath [45,53].

### 3.2. Suture Tape Augmentation/InternalBrace Ligament Augmentation

In a cohort study of adolescent patients (7–18 years old), 22 patients treated with STA were compared with 157 reconstruction patients [54]. The cumulative incidence of graft failure in the first three years after surgery was 48.8% (95% CI, 28.9–73.1%) in the STA group, as opposed to 4.7% (2.1–10.3%) in the reconstruction group. There was no difference in return to sports between the groups. Among individuals who did not rerupture their ACL, the PROMs as well as the range of motion were comparable between both groups. These results led to the conclusion that the high risk of failure for the STA group in this short-term follow-up should be considered when selecting the treatment for adolescent patients with an ACL injury. Ortmaier et al. matched 24 patients treated with STA with 25 hamstring and 20 quadriceps tendon reconstruction patients with a minimum follow-up of 12 months [55]. Overall, the return to sports rate was 91.3%. There were no significant differences in the number of sport disciplines and the return to sports time within or among the groups. Rerupture or repeat surgery rates are not mentioned. In a retrospective study of 27 patients with a mean age of 27.4 ± 8.6 years and a minimum of two years’ follow-up (range 2.0–3.8 years), a graft failure rate of 15% was reported [56]. Schneider et al. reported a revision surgery of 3% in a group of 88 STA patients with a mean age of 42 ± 13 years and a mean follow–up of 21 months. Patients’ age (>40 years), BMI (>30) and coexisting ligament or meniscal injuries did not seem to influence postoperative functional results [57]. Heusdens et al. published a prospective case report on their first 35 patients treated with STA with a two-year follow-up. Four patients (11.4%) suffered from a rerupture and three other patients (8.6%) needed a reintervention for another reason than rerupture. A preoperative Tegner score of ≥7 and grade 3 ACL healing on MRI at six months postoperatively were associated with a higher risk of rerupture [58].

### 3.3. Suture Anchor Primary ACL Repair

No results have been published yet by early adaptors of the SAPR technique. Achtnich et al. and Hoffmann et al. have performed ACL proximal repair with a comparable technique to the SAPR technique, but there are some differences [59,60]. Instead of two separate bundles, the ruptured ACL is reattached as one bundle and microfracturing is performed. Achtnich et al. describe in their case-control study comparable functional outcomes between 20 patients treated with proximal refixation of the ACL using knotless suture anchors and microfracturing versus 20 patients in the control group treated with single-bundle ACL recon [59]. Although the failure rate was 15% in the ACL refixation group and 0% in the reconstruction group, they suggest that refixation of the ACL is a feasible treatment option in carefully selected patients. Hoffmann et al. describe in their retrospective study on 12 patients with five years’ follow-up good to excellent clinical midterm outcomes in 75% of the patients [60]. Three patients (25%) experienced a failure. In cases of additional serious damage to extensor structures or systemic rheumatic disease, loss of function and unsatisfying clinical results occurred.

### 3.4. Bridge-Enhanced ACL Repair

No results have been published yet by early adaptors of the BEAR technique.

## 4. Present

One of the most important findings of the previously mentioned ACL repair manuscripts of the past decade is that the ACL is able to heal and subsequently restabilize the knee. ACL healing and subsequent knee stabilization has been proven clinically, during rearthroscopy and on MRI. Previously, it was thought that the ruptured ACL responds differently than the other knee ligaments and that it is not able to heal [9]. The continuous flow of synovial fluid in the knee hampers the formation of a stable fibrin–platelet clot between the ruptured ends of the ACL, which in turn will form stable scar tissue [12]. By bringing the ruptured ACL ends tight against each other (DIS/STA/SAPR) or by placing a bioactive scaffold to bridge the gap between the torn ligament ends (BEAR), the synovial fluid does not prevent the formation of stable scar tissue.

ACL repair could be a promising surgical technique with previously mentioned theoretical advantages over ACL recon. The question remains whether these advantages can be demonstrated in clinical practice and whether the midterm results will not deteriorate, as in the 1970s with the old ACL repair techniques. Can it be a game changer or will history repeat itself?

The four previously described ACL repair techniques show promising results published by the developers, which encouraged further research. Firstly, it was confirmed that the ACL is able to heal with modern arthroscopic ACL repair techniques. Secondly, the repaired ACL is able to stabilize the knee again, as measured with instrumented AP knee laxity. Thirdly, the rerupture rate of 0% to 10% for the first smaller case reports with two-year follow-up was promising. This rerupture rate increased to between 2.9% and 14% in larger studies, but was still reported as acceptable. Finally, PROMs were in the same range as ACL recon.

Through time, clinical results of early adaptors of the ACL repair techniques were published and the discussion became more diverse. Compared to the developers’ results, there seemed to be an overall higher rerupture rate and resurgery rate for other reasons than revision. Risk factors were described for failures and patient selection was emphasized. Risk factors for failure are a young age, high preinjury sports activity level, midsubstance ruptures, and impaired integrity of the ACL bundles and the synovial sheath. In a five-year follow-up study with 57 DIS patients Ahmad et al. underlined the potential of ACL repair, but also highlighted the danger of the procedure if strict patient selection is not appreciated [42]. In contrast, the higher rerupture and resurgery rates were not reflected in the three RCTs that have been published so far [34,51,52]. The two DIS versus ACL recon and the BEAR versus ACL recon RCTs with a two-year follow-up did not show a significant rerupture rate difference. The three RCTs reported a noninferiority or comparable results for PROMs for ACL repair compared to ACL recon.

The number of reviews on ACL repair is remarkable. In the past four years, 12 reviews have been published on ACL repair [61,62,63,64,65,66,67,68,69,70,71,72]. The overall consensus in these reviews is that prospective studies comparing ACL repair with ACL recon with sufficient follow-up are needed. Two reviews favor ACL recon over ACL repair [67,72]. Three reviews address the poor amount of high-quality evidence, which makes it difficult to establish the role of ACL repair [66,70,71]. The seven other reviews highlight the promising results or describe ACL repair as a (safe) treatment option for the acute ruptured ACL.

Currently the debate on ACL repair is continuing. The publications of the past few years taught us that the ruptured ACL is able to heal, but patient selection is critical.

## 5. Future

There are several issues that should be addressed in future ACL repair research. As mentioned in the ACL repair reviews, high-quality large RCTs between ACL recon and ACL repair, as well as between the different ACL repair techniques, are needed [73,74]. PROMs, return to work and sports, instrumented knee laxity, magnetic resonance imaging outcome, cost/utility analysis, reintervention for another reason than rerupture, and rerupture and failure rates and their risk factors should be addressed in these studies. As young patients (below the age of 25) and high-level athletes seem to have a higher risk of rerupture following ACL repair, possibly this subgroup is better treated with ACL recon. Although these groups have a higher risk on rerupture after ACL recon as well, the reported rerupture chance in the ACL repair case reports are higher (up to 44% at five years follow-up) [61,69]. The reported average age for an ACL rupture varies from 29.1 to 33.9—not only young and highly active teenagers rupture their ACLs [75,76]. In addition, proximal ACL ruptures are found more in the age group of 25 and older [29]. Several publications emphasize patient selection criteria of patients older than 25 and the non-high level athletes with an acute proximal bundle ACL rupture [58]. These patients could be the ideal candidates for ACL repair. That raises the question whether ACL repair is needed altogether for this group. Conservative treatment and rehabilitation under supervision of a dedicated physiotherapist is an underestimated treatment. Muaidi et al. describe in their systematic review a good short- to midterm prognosis in terms of self-reported knee function and functional performance after conservatively managed ACL-deficient knees [77]. However, subjects reduced their activity levels on average by 21% following injury. RCTs between conservative management, ACL repair and ACL recon could provide an answer for the patient group older than 25 and non-high athletes. A downside for conservative ACL treated patients with persistent instability is the diminished possibility for a successful ACL repair after 3–6 months.

Another interesting development is the improved understanding in the anterolateral complex [78]. ACL repair together with an anterolateral extra-articular procedure could reduce the rerupture rate. This could be especially interesting for patients younger than 25 and high-level athletes.

ACL reconstructed knees and nonoperatively treated knees demonstrated a 4.71 times and 2.41 times risk, respectively, for development of moderate to severe arthritis compared to controls [8]. ACL repair preserves the native insertion site as well the native ACL proprioceptors, which may in turn lead to more normal joint mechanics and decreased risk of post-traumatic osteoarthritis. [12] Long-term follow-up has to show if, in contrast to ACL recon or conservative treatment, ACL repair protects against the increased risk of post-traumatic osteoarthritis.

ACL recon still remains the gold standard until more ACL repair data can prove otherwise. Therefore, all ACL repair patients should be closely monitored and followed up, preferably in high-quality large RCTs.

## 6. Conclusions

ACL repair returned to the spotlight this decade. An important finding of the past decade is that the ACL is able to heal and subsequently restabilize the knee. Patient selection is emphasized: the ideal patient is a non-high athlete older than 25 and has an acute proximal one bundle ACL rupture. Future research will have to show if ACL repair could be a game changer or if history will repeat itself.

## Data Availability

Not applicable.

## References

[1] Feagin J.A., Curl W.W. (1976). Isolated tear of the anterior cruciate ligament: 5-year follow-up study. Am. J. Sports Med..

[2] Marshall J.L., Warren R.F., Wickiewicz T.L., Reider B. (1979). The anterior cruciate ligament: A technique of repair and reconstruction. Clin. Orthop. Relat. Res..

[3] Rousseau R., Labruyere C., Kajetanek C., Deschamps O., Makridis K.G., Djian P. (2019). Complications After Anterior Cruciate Ligament Reconstruction and Their Relation to the Type of Graft: A Prospective Study of 958 Cases. Am. J. Sports Med..

[4] Biau D.J., Tournoux C., Katsahian S., Schranz P.J., Nizard R.S. (2006). Bone-patellar tendon-bone autografts versus hamstring autografts for reconstruction of anterior cruciate ligament: Meta-analysis. BMJ.

[5] Sonnery-Cottet B., Saithna A. (2020). Editorial Commentary: Let’s ALL Agree-Anterior Cruciate Ligament Reconstruction Outcomes Need to Be Improved and Extra-Articular Procedures Have an Essential Role. Arthroscopy.

[6] Groot J.A., Jonkers F.J., Kievit A.J., Kuijer P.P., Hoozemans M.J. (2017). Beneficial and limiting factors for return to work following anterior cruciate ligament reconstruction: A retrospective cohort study. Arch. Orthop. Trauma Surg..

[7] Musahl V., Karlsson J. (2019). Anterior Cruciate Ligament Tear. N. Engl. J. Med..

[8] Anderson M.J., Browning W.M., Urband C.E., Kluczynski M.A., Bisson L.J. (2016). A Systematic Summary of Systematic Reviews on the Topic of the Anterior Cruciate Ligament. Orthop. J. Sports Med..

[9] Costa-Paz M., Ayerza M.A., Tanoira I., Astoul J., Muscolo D.L. (2012). Spontaneous healing in complete ACL ruptures: A clinical and MRI study. Clin. Orthop. Relat. Res..

[10] Steadman J.R., Matheny L.M., Briggs K.K., Rodkey W.G., Carreira D.S. (2012). Outcomes following healing response in older, active patients: A primary anterior cruciate ligament repair technique. J. Knee. Surg..

[11] Denti M., Monteleone M., Berardi A., Panni A.S. (1994). Anterior cruciate ligament mechanoreceptors. Histologic studies on lesions and reconstruction. Clin. Orthop. Relat. Res..

[12] Kiapour A.M., Murray M.M. (2014). Basic science of anterior cruciate ligament injury and repair. Bone Jt. Res..

[13] Kohl S., Evangelopoulos D.S., Kohlhof H., Hartel M., Bonel H., Henle P., von Rechenberg B., Eggli S. (2013). Anterior crucial ligament rupture: Self-healing through dynamic intraligamentary stabilization technique. Knee Surg. Sports Traumatol. Arthrosc..

[14] Kohl S., Evangelopoulos D.S., Ahmad S.S., Kohlhof H., Herrmann G., Bonel H., Eggli S. (2014). A novel technique, dynamic intraligamentary stabilization creates optimal conditions for primary ACL healing: A preliminary biomechanical study. Knee.

[15] Eggli S., Kohlhof H., Zumstein M., Henle P., Hartel M., Evangelopoulos D.S., Bonel H., Kohl S. (2015). Dynamic intraligamentary stabilization: Novel technique for preserving the ruptured ACL. Knee Surg. Sports Traumatol. Arthrosc..

[16] Henle P., Roder C., Perler G., Heitkemper S., Eggli S. (2015). Dynamic Intraligamentary Stabilization (DIS) for treatment of acute anterior cruciate ligament ruptures: Case series experience of the first three years. BMC Musculoskelet. Disord..

[17] Kohl S., Evangelopoulos D.S., Schar M.O., Bieri K., Muller T., Ahmad S.S. (2016). Dynamic intraligamentary stabilisation: Initial experience with treatment of acute ACL ruptures. Bone Jt. J..

[18] Heusdens C.H.W., Hopper G.P., Dossche L., Mackay G.M. (2018). Anterior Cruciate Ligament Repair Using Independent Suture Tape Reinforcement. Arthrosc. Tech..

[19] Heitmann M., Dratzidis A., Jagodzinski M., Wohlmuth P., Hurschler C., Puschel K., Giannakos A., Preiss A., Frosch K.H. (2014). Ligament bracing--augmented cruciate ligament sutures: Biomechanical studies of a new treatment concept. Der Unf..

[20] MacKay G., Anthony I.C., Jenkins P.J., Blyth M. (2015). Anterior Cruciate Ligament Repair Revisited. Preliminary Results of Primary Repair with Internal Brace Ligament Augmentation: A Case Series. Orthop. Muscular Syst. Curr. Res..

[21] Heusdens C.H.W., Hopper G.P., Dossche L., Roelant E., Mackay G.M. (2019). Anterior cruciate ligament repair with Independent Suture Tape Reinforcement: A case series with 2-year follow-up. Knee Surg. Sports Traumatol. Arthrosc..

[22] Proffen B.L., Vavken P., Haslauer C.M., Fleming B.C., Harris C.E., Machan J.T., Murray M.M. (2015). Addition of autologous mesenchymal stem cells to whole blood for bioenhanced ACL repair has no benefit in the porcine model. Am. J. Sports Med..

[23] DiFelice G.S., Villegas C., Taylor S. (2015). Anterior Cruciate Ligament Preservation: Early Results of a Novel Arthroscopic Technique for Suture Anchor Primary Anterior Cruciate Ligament Repair. Arthroscopy.

[24] DiFelice G.S., van der List J.P. (2018). Clinical Outcomes of Arthroscopic Primary Repair of Proximal Anterior Cruciate Ligament Tears Are Maintained at Mid-term Follow-up. Arthroscopy.

[25] van der List J.P., DiFelice G.S. (2016). Preservation of the Anterior Cruciate Ligament: A Treatment Algorithm Based on Tear Location and Tissue Quality. Am. J. Orthop. (Belle Mead NJ).

[26] van der List J.P., DiFelice G.S. (2016). Preservation of the Anterior Cruciate Ligament: Surgical Techniques. Am. J. Orthop. (Belle Mead NJ).

[27] van der List J.P., DiFelice G.S. (2017). Range of motion and complications following primary repair versus reconstruction of the anterior cruciate ligament. Knee.

[28] Jonkergouw A., van der List J.P., DiFelice G.S. (2019). Arthroscopic primary repair of proximal anterior cruciate ligament tears: Outcomes of the first 56 consecutive patients and the role of additional internal bracing. Knee Surg. Sports Traumatol. Arthrosc..

[29] van der List J.P., Jonkergouw A., van Noort A., Kerkhoffs G., DiFelice G.S. (2019). Identifying candidates for arthroscopic primary repair of the anterior cruciate ligament: A case-control study. Knee.

[30] Vermeijden H.D., Yang X.A., van der List J.P., DiFelice G.S. (2020). Role of age on success of arthroscopic primary repair of proximal anterior cruciate ligament tears. Arthroscopy.

[31] van der List J.P., Mintz D.N., DiFelice G.S. (2019). Postoperative Magnetic Resonance Imaging following Arthroscopic Primary Anterior Cruciate Ligament Repair. Adv. Orthop..

[32] van der List J.P., DiFelice G.S. (2018). Preoperative magnetic resonance imaging predicts eligibility for arthroscopic primary anterior cruciate ligament repair. Knee Surg. Sports Traumatol. Arthrosc..

[33] Proffen B.L., Sieker J.T., Murray M.M. (2015). Bio-enhanced repair of the anterior cruciate ligament. Arthroscopy.

[34] Murray M.M., Fleming B.C., Badger G.J., Freiberger C., Henderson R., Barnett S., Kiapour A., Ecklund K., Proffen B., Sant N. (2020). Bridge-Enhanced Anterior Cruciate Ligament Repair Is Not Inferior to Autograft Anterior Cruciate Ligament Reconstruction at 2 Years: Results of a Prospective Randomized Clinical Trial. Am. J. Sports Med..

[35] Haslauer C.M., Proffen B.L., Johnson V.M., Hill A., Murray M.M. (2014). Gene expression of catabolic inflammatory cytokines peak before anabolic inflammatory cytokines after ACL injury in a preclinical model. J. Inflamm. (Lond.).

[36] Haslauer C.M., Elsaid K.A., Fleming B.C., Proffen B.L., Johnson V.M., Murray M.M. (2013). Loss of extracellular matrix from articular cartilage is mediated by the synovium and ligament after anterior cruciate ligament injury. Osteoarthr. Cartil..

[37] Vavken P., Proffen B., Peterson C., Fleming B.C., Machan J.T., Murray M.M. (2013). Effects of suture choice on biomechanics and physeal status after bioenhanced anterior cruciate ligament repair in skeletally immature patients: A large-animal study. Arthroscopy.

[38] Proffen B.L., Fleming B.C., Murray M.M. (2013). Histologic Predictors of Maximum Failure Loads Differ between the Healing ACL and ACL Grafts after 6 and 12 Months In Vivo. Orthop. J. Sports Med..

[39] Murray M.M., Flutie B.M., Kalish L.A., Ecklund K., Fleming B.C., Proffen B.L., Micheli L.J. (2016). The Bridge-Enhanced Anterior Cruciate Ligament Repair (BEAR) Procedure: An Early Feasibility Cohort Study. Orthop. J. Sports Med..

[40] Murray M.M., Kalish L.A., Fleming B.C., Flutie B., Freiberger C., Henderson R.N., Perrone G.S., Thurber L.G., Proffen B.L., Ecklund K. (2019). Bridge-Enhanced Anterior Cruciate Ligament Repair: Two-Year Results of a First-in-Human Study. Orthop. J. Sports Med..

[41] Kösters C., Glasbrenner J., Raschke M., Lenschow S., Herbort M., Schliemann B. (2019). Clinical outcome 5 years after Dynamic Intraligamentary Stabilization of acute ACL ruptures. Orthop. J. Sports Med..

[42] Ahmad S.S., Schurholz K., Liechti E.F., Hirschmann M.T., Kohl S., Klenke F.M. (2019). Seventy percent long-term survival of the repaired ACL after dynamic intraligamentary stabilization. Knee Surg. Sports Traumatol. Arthrosc..

[43] Meister M., Koch J., Amsler F., Arnold M.P., Hirschmann M.T. (2018). ACL suturing using dynamic intraligamentary stabilisation showing good clinical outcome but a high reoperation rate: A retrospective independent study. Knee Surg. Sports Traumatol. Arthrosc..

[44] Osti M., El Attal R., Doskar W., Hock P., Smekal V. (2019). High complication rate following dynamic intraligamentary stabilization for primary repair of the anterior cruciate ligament. Knee Surg. Sports Traumatol. Arthrosc..

[45] Ateschrang A., Schreiner A.J., Ahmad S.S., Schroter S., Hirschmann M.T., Korner D., Kohl S., Stockle U., Ahrend M.D. (2019). Improved results of ACL primary repair in one-part tears with intact synovial coverage. Knee Surg. Sports Traumatol. Arthrosc..

[46] Heusdens C.H., Dossche L., Zazulia K., Michielsen J., Van Dyck P. (2019). Tips and Tricks to Optimize Surgical Outcomes After ACL Repair Using Dynamic Intraligamentary Stabilization. Surg. Technol. Int..

[47] Haberli J., Jaberg L., Bieri K., Eggli S., Henle P. (2018). Reinterventions after dynamic intraligamentary stabilization in primary anterior cruciate ligament repair. Knee.

[48] Benco M., Tylla A., Stangl R. (2019). Dynamic intraligamentary stabilization of acute anterior femoral cruciate ligament rupture: Preliminary and intermediate clinical results. Der Unf..

[49] Kosters C., Herbort M., Schliemann B., Raschke M.J., Lenschow S. (2015). Dynamic intraligamentary stabilization of the anterior cruciate ligament. Operative technique and short-term clinical results. Der Unf..

[50] Ateschrang A., Ahmad S.S., Stockle U., Schroeter S., Schenk W., Ahrend M.D. (2018). Recovery of ACL function after dynamic intraligamentary stabilization is resultant to restoration of ACL integrity and scar tissue formation. Knee Surg. Sports Traumatol. Arthrosc..

[51] Hoogeslag R.A.G., Brouwer R.W., Boer B.C., de Vries A.J., Huis In’t Veld R. (2019). Acute Anterior Cruciate Ligament Rupture: Repair or Reconstruction? Two-Year Results of a Randomized Controlled Clinical Trial. Am. J. Sports Med..

[52] Kosters C., Glasbrenner J., Spickermann L., Kittl C., Domnick C., Herbort M., Raschke M.J., Schliemann B. (2020). Repair With Dynamic Intraligamentary Stabilization Versus Primary Reconstruction of Acute Anterior Cruciate Ligament Tears: 2-Year Results From a Prospective Randomized Study. Am. J. Sports Med..

[53] Krismer A.M., Gousopoulos L., Kohl S., Ateschrang A., Kohlhof H., Ahmad S.S. (2017). Factors influencing the success of anterior cruciate ligament repair with dynamic intraligamentary stabilisation. Knee Surg. Sports Traumatol. Arthrosc..

[54] Gagliardi A.G., Carry P.M., Parikh H.B., Traver J.L., Howell D.R., Albright J.C. (2019). ACL Repair With Suture Ligament Augmentation Is Associated With a High Failure Rate Among Adolescent Patients. Am. J. Sports Med..

[55] Ortmaier R., Fink C., Schobersberger W., Kindermann H., Leister I., Runer A., Hepperger C., Blank C., Mattiassich G. (2020). Return to Sports after Anterior Cruciate Ligament Injury: A Matched-Pair Analysis of Repair with Internal Brace and Reconstruction Using Hamstring or Quadriceps Tendons. Sportverletz Sportschaden.

[56] Douoguih W.A., Zade R.T., Bodendorfer B.M., Siddiqui Y., Lincoln A.E. (2020). Anterior Cruciate Ligament Repair with Suture Augmentation for Proximal Avulsion Injuries. Arthrosc. Sports Med. Rehabil..

[57] Schneider K.N., Schliemann B., Gosheger G., Theil C., Weller J., Buddhdev P.K., Ahlbaumer G. (2020). Good to Excellent Functional Short-Term Outcome and Low Revision Rates Following Primary Anterior Cruciate Ligament Repair Using Suture Augmentation. J. Clin. Med..

[58] Heusdens C.H., Blockhuys K., Roelant E., Dossche L., Van Glabbeek F., van Dyck P. (2020). Suture Tape Augmentation ACL Repair; Stable Knee and Favorable PROMs, but a Re-rupture Rate of 11% Within Two Years. Knee Surg. Sports Traumatol. Arthrosc..

[59] Achtnich A., Herbst E., Forkel P., Metzlaff S., Sprenker F., Imhoff A.B., Petersen W. (2016). Acute Proximal Anterior Cruciate Ligament Tears: Outcomes After Arthroscopic Suture Anchor Repair Versus Anatomic Single-Bundle Reconstruction. Arthroscopy.

[60] Hoffmann C., Friederichs J., von Ruden C., Schaller C., Buhren V., Moessmer C. (2017). Primary single suture anchor re-fixation of anterior cruciate ligament proximal avulsion tears leads to good functional mid-term results: A preliminary study in 12 patients. J. Orthop. Surg. Res..

[61] Kandhari V., Vieira T.D., Ouanezar H., Praz C., Rosenstiel N., Pioger C., Franck F., Saithna A., Sonnery-Cottet B. (2020). Clinical Outcomes of Arthroscopic Primary Anterior Cruciate Ligament Repair: A Systematic Review from the Scientific Anterior Cruciate Ligament Network International Study Group. Arthroscopy.

[62] Malahias M.A., Chytas D., Nakamura K., Raoulis V., Yokota M., Nikolaou V.S. (2018). A Narrative Review of Four Different New Techniques in Primary Anterior Cruciate Ligament Repair: “Back to the Future” or Another Trend?. Sports Med. Open.

[63] Mahapatra P., Horriat S., Anand B.S. (2018). Anterior cruciate ligament repair-past, present and future. J. Exp. Orthop..

[64] Bucci G., Moatshe G., Lebus G.F., Singleton S.B. (2020). Arthroscopic Primary Repair of the Anterior Cruciate Ligament: A Narrative Review of the Current Literature. Knee Surg Sports Traumatol. Arthrosc..

[65] van der List J.P., Vermeijden H.D., Sierevelt I.N., DiFelice G.S., van Noort A., Kerkhoffs G. (2019). Arthroscopic primary repair of proximal anterior cruciate ligament tears seems safe but higher level of evidence is needed: A systematic review and meta-analysis of recent literature. Knee Surg. Sports Traumatol. Arthrosc..

[66] Papalia R., Torre G., Papalia G., Campi S., Maffulli N., Denaro V. (2019). Arthroscopic primary repair of the anterior cruciate ligament in adults: A systematic review. Br. Med Bull..

[67] Nwachukwu B.U., Patel B.H., Lu Y., Allen A.A., Williams R.J. (2019). Anterior Cruciate Ligament Repair Outcomes: An Updated Systematic Review of Recent Literature. Arthroscopy.

[68] van Eck C.F., Limpisvasti O., ElAttrache N.S. (2018). Is There a Role for Internal Bracing and Repair of the Anterior Cruciate Ligament? A Systematic Literature Review. Am. J. Sports Med..

[69] Ahmad S.S., Schreiner A.J., Hirschmann M.T., Schroter S., Dobele S., Ahrend M.D., Stockle U., Ateschrang A. (2019). Dynamic intraligamentary stabilization for ACL repair: A systematic review. Knee Surg. Sports Traumatol. Arthrosc..

[70] Hoogeslag R.A.G., Brouwer R.W., de Vries A.J., Boer B.C., Huis In ‘t Veld R. (2020). Efficacy of Nonaugmented, Static Augmented, and Dynamic Augmented Suture Repair of the Ruptured Anterior Cruciate Ligament: A Systematic Review of the Literature. Am. J. Sports Med..

[71] Anterior Cruciate Ligament Injury: Is ACL Repair Better Than ACL Reconstruction?. https://myorthoevidence.com/Blog/show/97.

[72] Hughes J.D., Lawton C.D., Nawabi D.H., Pearle A.D., Musahl V. (2020). Anterior Cruciate Ligament Repair: The Current Status. J. Bone Jt. Surg Am..

[73] Engebretsen L. (2020). Editorial Commentary: The Anterior Cruciate Ligament Cannot be Reliably Repaired: Studies With a Control Group are Needed!. Arthroscopy.

[74] Heusdens C.H.W., Zazulia K., Roelant E., Dossche L., van Tiggelen D., Roeykens J., Smits E., Vanlauwe J., Van Dyck P. (2019). Study protocol: A single-blind, multi-center, randomized controlled trial comparing dynamic intraligamentary stabilization, internal brace ligament augmentation and reconstruction in individuals with an acute anterior cruciate ligament rupture: LIBR study. BMC Musculoskelet. Disord..

[75] Sanders T.L., Maradit Kremers H., Bryan A.J., Larson D.R., Dahm D.L., Levy B.A., Stuart M.J., Krych A.J. (2016). Incidence of Anterior Cruciate Ligament Tears and Reconstruction: A 21-Year Population-Based Study. Am. J. Sports Med..

[76] Nicholls M., Aspelund T., Ingvarsson T., Briem K. (2018). Nationwide study highlights a second peak in ACL tears for women in their early forties. Knee Surg. Sports Traumatol. Arthrosc..

[77] Muaidi Q.I., Nicholson L.L., Refshauge K.M., Herbert R.D., Maher C.G. (2007). Prognosis of conservatively managed anterior cruciate ligament injury: A systematic review. Sports Med..

[78] Getgood A., Brown C., Lording T., Amis A., Claes S., Geeslin A., Musahl V. (2019). The anterolateral complex of the knee: Results from the International ALC Consensus Group Meeting. Knee Surg. Sports Traumatol. Arthrosc..

